# Structural Changes of RNA in Complex with Proteins in the SRP

**DOI:** 10.3389/fmolb.2018.00007

**Published:** 2018-02-05

**Authors:** Janine K. Flores, Sandro F. Ataide

**Affiliations:** Ataide Lab, School of Life and Environmental Sciences, University of Sydney, Sydney, NSW, Australia

**Keywords:** RNA structure, protein conformation, RNP complexes, RNA interaction, RNA structure and function

## Abstract

The structural flexibility of RNA allows it to exist in several shapes and sizes. Thus, RNA is functionally diverse and is known to be involved in processes such as catalysis, ligand binding, and most importantly, protein recognition. RNA can adopt different structures, which can often dictate its functionality. When RNA binds onto protein to form a ribonucleoprotein complex (RNP), multiple interactions and conformational changes occur with the RNA and protein. However, there is the question of whether there is a specific pattern for these changes to occur upon recognition. In particular when RNP complexity increases with the addition of multiple proteins/RNA, it becomes difficult to structurally characterize the overall changes using the current structural determination techniques. Hence, there is a need to use a combination of biochemical, structural and computational modeling to achieve a better understanding of the processes that RNPs are involved. Nevertheless, there are well-characterized systems that are evolutionarily conserved [such as the signal recognition particle (SRP)] that give us important information on the structural changes of RNA and protein upon complex formation.

## Introduction

Upon its discovery, RNA was initially confined as the essential component in gene expression and protein synthesis (the classical view of RNA as transfer RNA, ribosomal RNA, and messenger RNA). Nowadays RNA is known to have a central role in widespread functional complexes throughout the cell with most, if not all, RNA being part of processes essential for cell survival (e.g., long non-coding RNA, micro RNA, and many more; Draper, 1999; Kligun and Mandel-Gutfreund, 2015; Blythe et al., 2016; Schlundt et al., 2017). Many of these RNAs function by forming RNA–protein or ribonucleoprotein (RNP) complexes, which can contain multiple protein subunits bound to one or more RNA molecules. Many of RNPs have essential biological function in processes such as the regulation of transport, localization, splicing, and RNA processing/regulation (Schlundt et al., 2017). Hence, understanding the molecular mechanism and structural features of these complexes is a fundamental need in science. To date only a handful of RNPs have been studied in large detail, providing the prime examples of RNP complexes involved in crucial processes [the spliceosome (Zhang et al., 2017), the ribosome (Ramakrishnan, 2002; Khatter et al., 2015), RNA polymerase (Hahn, 2004; Carter and Drouin, 2009), and the signal recognition particle (SRP; Kuglstatter et al., 2002; Hainzl et al., 2007; Ataide et al., 2011; Grotwinkel et al., 2014; Becker et al., 2017)]. In this review, we summarize the overall RNA conformational changes and interactions that occur upon its binding to proteins. We focus on an evolutionarily conserved RNP known as the SRP to demonstrate how some structural features are conserved across all kingdoms of life.

## RNP complex formation

**Figure 1:**
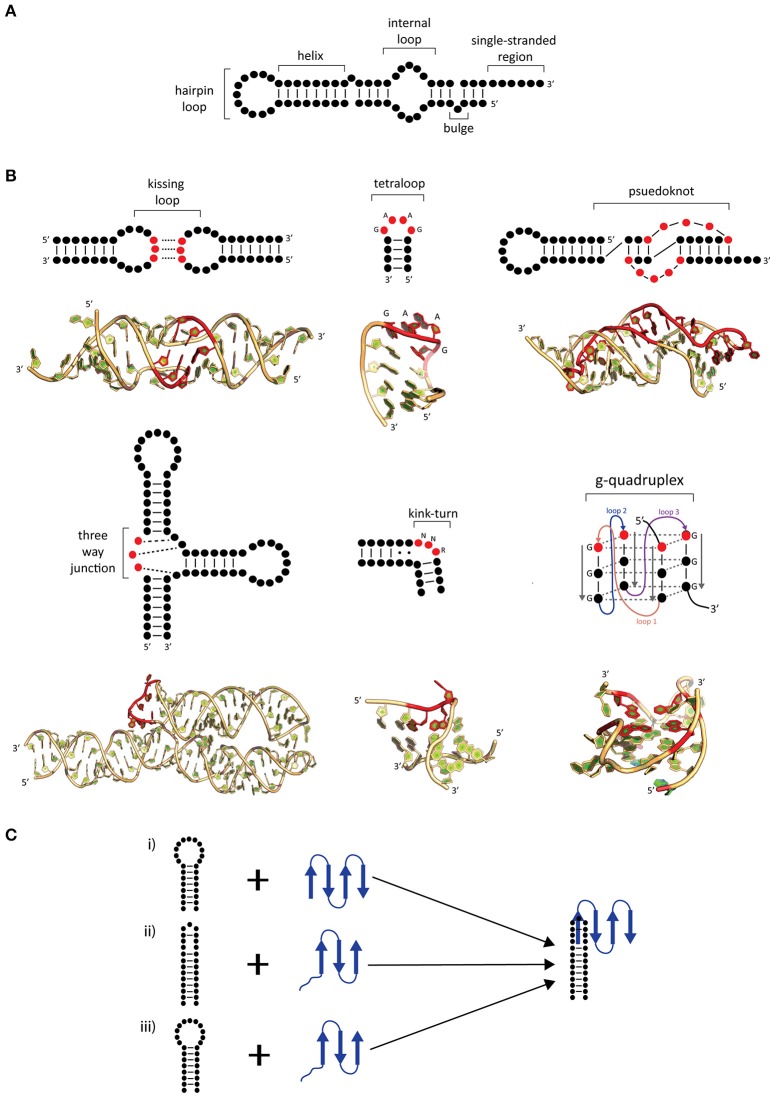
Common secondary and tertiary motifs of RNA and RNA conformational changes in RNP complexes. **(A)** 2-D representations of common RNA secondary motifs, **(B)** 2-D representations of common RNA secondary and tertiary structural motifs with 3-D examples of crystal structures. A kissing loop structure from HIV-1 dimerization (PDB: 1K9W; Ennifar et al., 2001). A GAAG tetraloop from SRP RNA (PDB: 2F87; Okada et al., 2006). A telomerase pseudoknot (PDB: 2K96; Kim et al., 2008). A three-way junction 7S RNA (PDB: 1MFQ; Kuglstatter et al., 2002). A kink-turn found from SAM-I riboswitch (PDB: 3IQN; Stoddard et al., 2010) and g quadruplex site from telomeric RNA (PDB: 31BK; Collie et al., 2010). Highlighted bases (red) show characteristic features of these RNA tertiary structures. **(C)** Possible mechanisms of induced fit **(i–iii)** and conformational capture **(i–ii)**. The RNA (black) and protein (blue) form a complex by either **(i)** protein-induced RNA folding, **(ii)** RNA-induced protein folding, or **(iii)** mutual folding of the RNA and protein.

RNA, in comparison to DNA, is more flexible and can exist in a variety of secondary and tertiary (3D) motifs (Batey et al., 1999; Butcher and Pyle, 2011; Jones and Ferré-D'Amaré, 2015; Blythe et al., 2016). RNA secondary motifs can range from double stranded helices, loops, junctions, and bulges of single-stranded regions (Figure 1A; Blythe et al., 2016). Upon folding, RNA can adopt several tertiary motifs to maximize base stacking through co-axial stacking of adjacent helices and other structures, stabilizing its 3D motif (Butcher and Pyle, 2011; Blythe et al., 2016). Additionally, RNA 3D motifs are formed through interactions between secondary structural motifs (such as kissing loops, pseudoknots, kink turns, tetraloops, and g-quadruplexes; Figure 1B; for review, see Batey et al., 1999; Butcher and Pyle, 2011). The coaxial stacking of helices, along with sequence specific interactions, the formation of base stacking and backbone interactions upon folding are major determinants of the overall RNA architecture. While RNA folding is a thermodynamically favored process incurring an increase in free energy (ΔG) in the system, the binding of proteins onto RNA assists in achieving the active 3D fold (Herschlag, 1995; Williamson, 2000). Furthermore, the overall RNA folding and RNP formation protects the RNA backbone from hydroxyl radicals minimizing RNA degradation (Schroeder et al., 2004).

RNA-binding motifs for RNA-binding proteins such as RNA Recognition Motifs, (RRMs), double-stranded RNA Binding Domains (dsRBDs), K-Homology (KH) domains, and Zinc Fingers (ZF) and their modes of binding onto RNA have been extensively studied. However, information on the contribution of RNA to the overall binding of the complex is lacking in comparison to the protein counterparts (Lunde et al., 2007; Khatter et al., 2015; Schlundt et al., 2017). The combinatorial effect of multiple RNA 3D motifs and protein binding motifs makes it difficult to deduce the binding mechanism of RNA onto protein(s). This effect is enhanced as it can vary significantly from complex-to-complex depending on the type/structure of RNA. Furthermore, additional RNA post-transcriptional modifications can either stabilize or disrupt structural elements and influence protein accessibility (e.g., tRNA, rRNA, and mRNA) increasing the variability in complex formation (Batey et al., 1999; Natchiar et al., 2017). Thus, there is difficulty in understanding the overall mechanisms in which RNA interacts with protein to form RNPs. A combined effort of structures, biochemical information, molecular dynamics simulation (MD) and computational analysis becomes necessary to understand and manipulate RNPs due to the large variability in RNP complex formation, conformational changes, and interactions between RNA and protein.

### Conformational changes upon RNP formation

The mechanism generalization of RNA–protein binding has been hindered due to the large and diverse number of interactions between them. Interaction between RNA–protein comprises the core feature of RNP complex formation and often accompanied by conformational changes in either or both RNA and protein. Comparisons of X-ray structures between *apo* proteins and RNA with the RNP have shown that there are several possible mechanisms that can be used to describe the conformational changes of RNP complexes. These mechanisms are known as induced fit and conformational capture (Figure 1C; Williamson, 2000; Leulliot and Varani, 2001; Qin et al., 2010).

In induced fit, the RNA and protein individually or as a complex undergo drastic conformational changes upon binding. This occurs when there is flexibility in either component, resulting in an increase in affinity and specificity (Williamson, 2000; Leulliot and Varani, 2001) and may occur in one of two ways. Firstly, the RNA may undergo substantial change upon protein binding (Figure 1Ci), as is the case with of ribosomal S15 bound onto rRNA. In comparison to the structures of *apo* rRNA (Orr et al., 1998) and S15 protein (Clemons et al., 1998), it was found in the S15-rRNA crystal structure that there is a large conformational change in the rRNA causing two helices to coaxially stack and the third to form an acute angle from its original 120° upon S15 binding (Agalarov et al., 2000; Nikulin et al., 2000). The opposite can also occur where the protein undergoes a significant change in comparison to RNA (Figure 1Cii; Williamson, 2000; Pérez-Cano et al., 2017). Secondly, the RNA and protein may mutually undergo conformational changes upon complex formation (Figure 1Ciii). An example of mutually induced fit is the U1A-UTR complex that is involved in the regulation of polyadenylation. The binding of the UTR RNA and U1A protein (Avis et al., 1996; Gubser and Varani, 1996) causes the RNA to stack which allows for the RNA and protein to pack more closely together as deduced from the U1A-RNA crystal structure (Oubridge et al., 1994).

In conformational capture (also known as conformational selection or tertiary structure capture), the RNA or protein will only recognize a specific conformation of its binding partner to form a complex (Leulliot and Varani, 2001). This is demonstrated between the phage R17 protein, MS2, and a hairpin from its genome (Draper, 1999). In MS2-RNA recognition, the protein recognizes adenine (A) in positions 4 and 10 in the RNA hairpin, which are in the correct spatial orientation. Upon recognition of these bases the protein will then bind onto the RNA by forming hydrogen bonds between A10 and Lys61/Val29 on MS2. It is known that substitutions to A10 to a pyrimidine (C, U) can significantly decrease binding due to the loss of hydrogen bonding contacts (Draper, 1999). Thus, the RNA hairpin is the conformation being “captured” to form a complex. After the conformational selection of the RNA, the protein must then undergo a conformational change to form an “induced fit” resulting in a functional complex. Overall, induced fit and conformational capture are intrinsically linked mechanisms with the latter occurring only if the bound conformation pre-exists as a minor population in *apo* form in the cell prior to the binding of its partner.

### RNA sequence-specific and non-specific interactions

RNP complex formation also requires an intricate combination of interactions between the RNA and protein. Based on several structural studies using X-ray crystallography, nuclear magnetic resonance spectroscopy (NMR), and cryo electron microscopy (Cryo-EM), computational modeling (Patel et al., 2017; Pérez-Cano et al., 2017) and database analysis of the structures in the PDB (Jones et al., 2001; Treger and Westhof, 2001; Jeong et al., 2003; Ellis et al., 2007; Bahadur et al., 2008; Gupta and Gribskov, 2011; Jones, 2016) some relationship between RNAs and how they bind to protein have been identified. RNA and protein binding can be classified as either base-specific, protein side-chain specific or non-specific interactions (Jones et al., 2001; Treger and Westhof, 2001; Jeong et al., 2003; Gupta and Gribskov, 2011; Iwakiri et al., 2012; Pérez-Cano et al., 2017).

RNA specificity occurs due to the flexibility of the RNA backbone (due to the formation of loops and bulges in RNA secondary motifs and subsequent tertiary motifs upon folding) and bases (purines—A, G and pyrimidines—C, U), which allows for the specific recognition sites on the RNA to be exposed. The specific site recognition is mediated by hydrogen bonding or stacking interactions with protein side chains (Draper, 1999; Kligun and Mandel-Gutfreund, 2015). In terms of specificity, a statistical study by Gupta and Gribskov (2011) between several RNA–protein structures in the PDB has shown that 24.6% of RNA–protein interactions (in particular hydrogen bonds) are base-specific. An example is the U1A protein, which recognizes the AUUGCAC motif when it's either in a hairpin loop or an internal loop of the RNA, showing little preference for structure (Draper, 1999).

However, most of these interactions are non-sequence specific (75.4%; Gupta and Gribskov, 2011; Pérez-Cano et al., 2017). Depending on the type of interactions, there are trends in the protein binding to RNA with guanine (G) being preferred in base-specific interactions and disfavored in non-specific interactions (Gupta and Gribskov, 2011). Other statistical analyses on the shape of the protein surface have shown that protruded surfaces usually form electrostatic interactions with the backbone of RNA whereas dented surfaces form hydrogen bonds in between the protein backbone and RNA base (Gupta and Gribskov, 2011; Iwakiri et al., 2012; Kligun and Mandel-Gutfreund, 2015) showing the importance of protein structural states upon binding. However, as for conformational changes in RNP complexes, RNA–protein interactions can involve a combination of specific or non-specific interactions. Comparative studies of structures in the PDB have shown that not only is the RNA and protein sequence important in recognition but contribute significantly to the binding (Gupta and Gribskov, 2011; Iwakiri et al., 2012). Strikingly the structure of both the RNA and protein are more important than the sequence specificity for their binding with a clear bias toward unpaired or single-stranded RNA regions (Ellis et al., 2007; Gupta and Gribskov, 2011; Kligun and Mandel-Gutfreund, 2015). Thus, more information on RNP structure are needed to allow us to better predict RNA–protein binding interfaces.

## Structural determination of RNPs

In the Protein Data Bank (PDB), there is a continuously growing number of *apo* protein, protein-DNA and protein–protein complex structures determined by X-ray crystallography, NMR, and cryo-EM (Bernstein et al., 1978). However, with *apo* RNA and RNPs the number of available structures is limited due to the difficulties in solving their structures (Jones, 2016; Patel et al., 2017). As of October 2017, there are now 1,582 X-ray crystallography structures, 117 NMR structures, 377 EM structures, and 6 fiber diffraction structures released on the PDB of RNA or RNPs. Although these numbers are small in comparison to non-RNP X-ray structures (~119,000), there is some information that can be learnt when comparing the unbound structures of the protein and the RNA to RNP complexes. Recently, MD simulations and computational docking of protein-RNA complexes using available *apo* structures has provided us with further information on how RNA may bind protein. However, due to the lack of available RNP structures, this technique requires more optimization to ensure proper parameters are available for modeling (Jones, 2016; Patel et al., 2017; Pérez-Cano et al., 2017).

### Limitations of RNP structural determination

While crystallography has been the strongest and preferred method to delineate these interactions and conformational changes, there are several limitations and challenges in using this technique (Flores et al., 2014). Firstly, crystallography can only capture a singular homogenous conformation that must crystallize (Leulliot and Varani, 2001; Ellis and Jones, 2008). This limits our understanding of the intermediate processes that are involved in complex formation. In particular, when several conformational changes occur within the RNA upon binding of several proteins (Williamson, 2000). Secondly, a resolution of 3 Å or higher is often necessary to accurately determine and identify the interactions between protein and RNA (Gupta and Gribskov, 2011; Jones, 2016). A further limitation of the use of crystallography comes from truncations made in proteins and RNA due to the difficulties in crystallizing large RNP complexes yielding information to only part of the complex (Jones, 2016; Schlundt et al., 2017). Despite the limiting number of *apo* and RNP structures available from crystallography, NMR, and cryo-EM in the databases, they provide a fundamental partial knowledge of the conformational changes and interactions that occur in RNP formation. Although these are some of the limitations of using crystallography to obtain structural information for RNP complexes, all other techniques have unique difficulties. For example, structural determination via NMR have molecular weight boundaries that confine the technique to relatively small complexes, whereas cryo-EM requires extensive sample preparation and optimization of large complexes. Nonetheless, a combination of these structural techniques along with computational and biochemical methods may provide vital information that is fundamental to our understanding of the biological processes in which RNA is involved.

## Multi-subunit RNP complex formation

Similar to protein–protein and DNA–protein complexes, all components of a RNP complex must pass through multiple energy barriers in order to become a functional macromolecule. A folding protein tends to adopt its optimal conformation and minimal energy quickly, however an RNP composed by one or more proteins, and an RNA with its multiple conformers, has several energy barriers that must be overcome. Sometimes the binding events must follow a very specific order (e.g., ribosome and SRP biogenesis) to achieve an active state. While protein folding and secondary structure prediction (e.g., JPred4, Phyre2, and PSIPRED) are well-established methods, predicting RNA fold (mfold and viennaRNA) becomes more complex as its size increases (e.g., long non-coding RNAs, >200 nt). An example is the human SRP where crystal structures of SRP19-7S RNA (Oubridge et al., 2002) and SRP19-SRP54-7S RNA (Kuglstatter et al., 2002) have shown that SRP19 must bind first onto helix 6 and 8 to bring these closer together for SRP54 to bind properly (Maity et al., 2006). In this case, a combination of conformational capture and induced fit are involved in the formation of this complex with SRP19 recognizing the tetraloop. Thus, structural analyses of RNP complexes in different states of assembly are essential in order to understand the interactions and conformational changes. To gain an understanding of RNPs, the SRP will be used as an example of the changes that occur during multi-protein RNP complex formation.

## Evolutionarily conserved RNPs and its RNA

Looking across all kingdoms of life we can identify several conserved RNPs and learn about evolution with them, specially with the few examples where multiple structures from different kingdoms have been solved (e.g., the ribosome and the SRP; Steitz, 2008). A key feature is the increased complexity and size of RNPs in eukaryotes compared with bacteria or Achaea. This poses the question of whether the core structure and interactions in the RNPs can provide information about structure and function of the RNA and proteins.

### SRP in all domains of life

The SRP was discovered by Blobel et al. (1979) (see reviews: Rupert and Ferré-D'Amaré, 2000; Doudna and Batey, 2004; Akopian et al., 2013) where its primary function is to recognize the N-terminal hydrophobic signal sequence of the nascent peptide at the exit tunnel of a translating ribosome and deliver it to a translocon (Doudna and Batey, 2004). SRP delivers proteins to the endoplasmic reticulum in eukaryotes and to plasma membrane in archaea and bacteria (Rosenblad et al., 2009). SRP composition varies to include six proteins (SRP9/14, SRP19, SRP54, and SRP68/72) and a long-noncoding RNA in eukaryotes, two proteins (SRP19, SRP54), and its RNA in archaea and one protein [fifty-four homolog (Ffh)] and RNA in bacteria. Archaeal and eukaryotic SRP systems are also further subdivided into two functional domains known as the Alu and S domains. SRP RNA along with SRP54 (in eukaryotes and archaea) or Ffh (in bacteria) are the core components of all SRP complexes (Rupert and Ferré-D'Amaré, 2000).

### Conserved SRP RNA structural features and interactions

**Figure 2:**
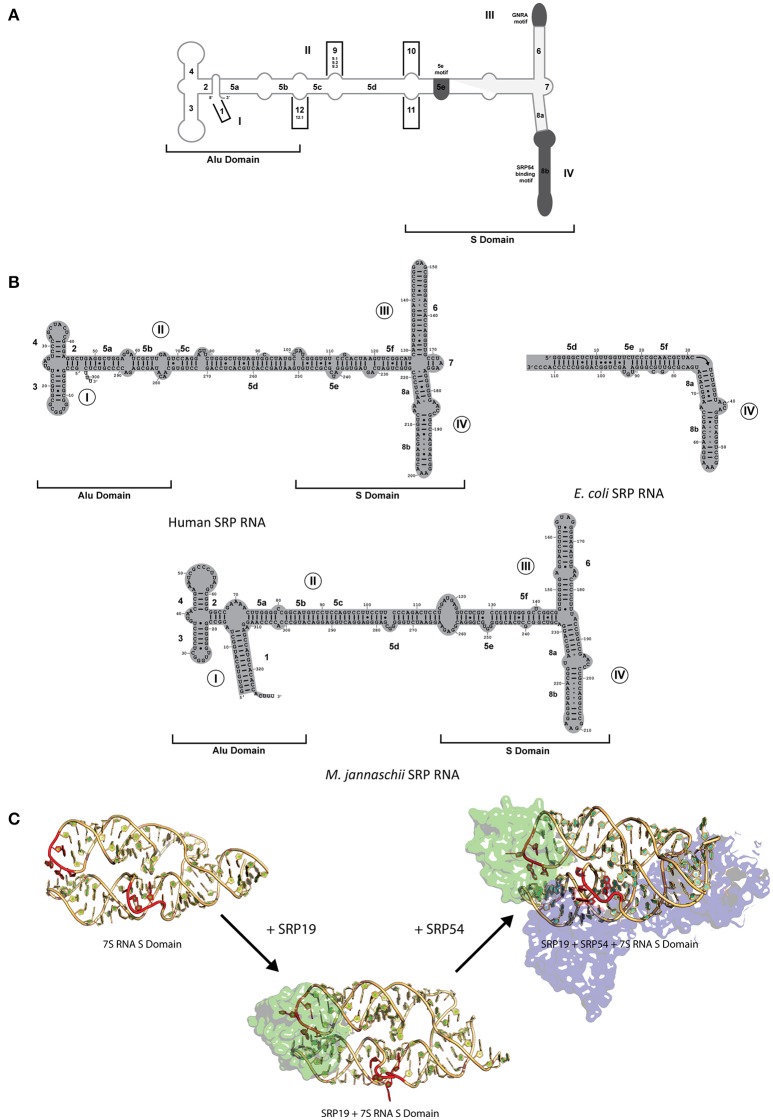
SRP RNA secondary structures and conformational changes during SRP RNP formation. **(A)** SRP nomenclature. Mammalian SRP RNA secondary structure is traced in gray. Common motifs and helices are colored in dark gray. Helices are numbered 1–12 with helical sections labeled a–f. Domains are labeled I–IV. The approximate boundaries of the Alu domain and S domain are labeled. 5′ and 3′ ends are indicated **(B)** Secondary structures or the eukaryotic, bacterial and archaeal SRP RNA. Examples are shown of the eukaryotic (human), bacterial (*Escherichia coli*), and archaeal (*Methanococcus jannaschii*). Helices are numbered 1–8 with helical sections labeled a–f. Residues are numbered in increments of 10. Domains are labeled I–IV. The approximate boundaries of the Alu domain and S domain of the eukaryotic and archaeal SRP RNAs are labeled. 5′ and 3′ ends are indicated. **(C)** RNA conformational changes upon SRP complex formation in *M. jannaschii*. Crystal structure of apo 7S RNA of the S domain is shown (PDB: 1Z43). Crystal structures of SRP19 and 7S RNA S domain (PBD: 1LNG) and SRP19 and 7S RNA S domain (PBD: 2V3C) are shown. RNA conformational changes upon SRP19 binding and subsequent binding of SRP54 are shown in red.

SRP RNA is one critical component of the SRP complex present in all domains of life with its size and secondary structure varying considerably between organisms (Rosenblad et al., 2009). SRP RNA is composed of several helices (1–12), domains (I–IV), and motifs (5e motif, GNAR tetraloop; Figure 2A). Only domain IV which is part of helix 8 is present in all kingdoms of life (Figure 2B; Schmitz et al., 1999; Zwieb et al., 2005) and contain a conserved asymmetric loop in helices 8a and 8b with the latter containing an invariant A-C pair and a highly conserved non-canonical G-G and G-A pair (Rupert and Ferré-D'Amaré, 2000; Rosenblad et al., 2009). The M-domain of SRP54/Ffh binds to this region of the SRP RNA through an induced fit mechanism, where the asymmetric loop presents a 5′ adenosine that is recognized by three conserved amino acids (Arg398, Arg401, and Glu386 in *Escherichia coli* based on Batey et al., 2000; Figure 2C; Batey et al., 2000; Bernstein, 2000; Doudna and Batey, 2004). Crystal structures of the complex in comparison to NMR structures of the RNA have shown that the RNA undergoes a significant structural rearrangement while SRP54/Ffh maintains its structure upon binding (Figure 2C; Rupert and Ferré-D'Amaré, 2000). This interaction is clearly mediated by a combination of sequence and structure specificity of the RNA.

While SRP54/Ffh and SRP RNA are the only conserved components between all three kingdoms, archaeal and eukaryotic SRP also share a common protein, SRP19 (Doudna and Batey, 2004). The SRP RNA between archaea and eukaryotes have some differences in structure (both contain helices 2–6 and 8 with archaea lacking helix 7 and eukaryotes lacking helix 1) and are similar in size in comparison to the smaller bacterial SRP RNA (containing helices 5 and 8; Figure 2B; Zwieb et al., 2005; Rosenblad et al., 2009). Interestingly, in terms of conformational changes, the complex assembles through an induced fit mechanism through the movement of domain III and IV on the SRP RNA (Rose and Weeks, 2001; Kuglstatter et al., 2002; Hainzl et al., 2005). It has been observed crystallographically and biochemically that SRP19 binds through the recognition of apical tetraloops of helices 6 and 8 (Figure 2C; Kuglstatter et al., 2002; Hainzl et al., 2005; Rosenblad et al., 2009). Stabilization of the SRP RNA structure occurs upon binding of SRP19 through the asymmetric internal loop motif of helix 8 which forms two A-minor motifs with helix 6 (Kuglstatter et al., 2002; Doudna and Batey, 2004). This increase the stabilization of the SRP RNA structure upon formation of additional RNA–RNA contacts that allows the cooperative effect in assembly and subsequent binding of SRP54 to form a functional complex in both archaea and eukaryotes (Rose and Weeks, 2001; Kuglstatter et al., 2002; Doudna and Batey, 2004; Hainzl et al., 2005). Further movement on the human RNA may also occur due to the presence of the Alu domain and the binding of heterodimers, SRP9/14 and SRP68/72, onto the RNA (Weichenrieder et al., 2000). Despite the differences in the SRPs complexity between organisms, the interactions, and conformational changes of these regions of the SRP RNA and protein(s) involved have been evolutionarily conserved to achieve the optimal active structure.

## Conclusion

The further we understand the molecular interactions and structural changes that takes place in the RNA and protein counterparts in RNP complex formation, the more evident it becomes that RNPs do not behave as the canonical binding observed in protein–protein and protein–DNA interactions. RNA interactions, and a greater level of fluid conformational changes, play a large role in these complexes formation and there is much more to be understood. In the past, crystallography has played a large role in identifying and visualizing the interactions of these conformational changes through the structural determination of the *apo* RNA/protein components and its RNP complexes. However, despite all the limitations, a combined use of NMR and cryo-EM will allow us to further observe these interactions and structural changes the RNA undergoes in the formation of RNPs. Further atomic and structural information from these techniques along with the use of MD simulations and computational docking can be extremely beneficial in the field especially with much larger complexes.

## Author contributions

JF and SA wrote the review together.

### Conflict of interest statement

The authors declare that the research was conducted in the absence of any commercial or financial relationships that could be construed as a potential conflict of interest.
